# CO_2_ Transoral Microsurgery for Supraglottic Squamous Cell Carcinoma

**DOI:** 10.3389/fonc.2018.00321

**Published:** 2018-09-04

**Authors:** Filippo Carta, Cinzia Mariani, Giovanni B. Sambiagio, Natalia Chuchueva, Elisa Lecis, Clara Gerosa, Roberto Puxeddu

**Affiliations:** ^1^Unit of Otorhinolaryngology, Department of Surgery, Azienda Ospedaliero-Universitaria di Cagliari, University of Cagliari, Cagliari, Italy; ^2^NHS Lanarkshire, Glasgow, United Kingdom; ^3^I.M. Sechenov First Moscow State Medical University, Moscow, Russia; ^4^Unit of Pathology, Department of Surgery, Azienda Ospedaliero-Universitaria di Cagliari, University of Cagliari, Cagliari, Italy

**Keywords:** supraglottic carcinoma, endoscopic surgery, CO_2_ laser, swallowing, microlaryngoscopy, functional results

## Abstract

The present study analyzed the results of the endoscopic approach to T1, T2 and selected T3 supraglottic carcinoma with the aim of reviewing functional and oncologic outcomes after different types of endoscopic supraglottic laryngectomies. This is a retrospective clinical study of 42 consecutive patients (mean age of 61.8 years, 33 males, 9 females) treated by the senior author for supraglottic squamous cell carcinoma with a transoral CO_2_ laser approach and reviewed from November 2010 to September 2017. Surgical procedures were classified according to the European Laryngological Society. In addition to the standardized transoral supraglottic laryngectomies, we introduced a modified type IVb by sparing the inferior third of the arytenoid if not directly involved in the tumor. Swallowing was evaluated with the Swallowing Performance Status Scale reported by the Multinational Association of Supportive Care in Cancer/International Society of Oral Oncology. Survival probabilities were estimated using Kaplan-Meier curves. Two type I, 2 type IIa, 2 type IIb, 3 type IIIa, 12 type IIIb, 13 type IVa, 3 type modified IVb, and 5 type IVb supraglottic laryngectomies were performed. Twenty-one patients (50%) underwent primary neck dissection. The pathologic TNM classification according to the 8th edition of the American Joint Committee on Cancer system was as follows: 9 pT1cN0, 2 pT1N0, 1 pT1N1, 7 pT2cN0, 1 rypT2cN0, 9 pT2N0, 4 pT2N1, 2 ypT2N1, 2 pT3cN0, 2 rypT3cN0, 1 pT3N1, and 2 pT3N2b. Mean follow-up was 3.4 years (range of 9 months to 6 years). According to the Kaplan–Meier analysis, 5-year disease-specific survival, local-relapse-free survival, nodal-relapse-free survival, overall laryngeal preservation and overall survival of patients without previous head and neck radiotherapy/open surgery were 100%, 95.2%, 87.8%, 100%, and 64.6%, respectively. Patients who underwent type I, IIa, and IIb resections (*n* = 6) started oral feeding the day after surgery, patients who underwent type III-IVb modified resections (*n* = 31) started oral feeding 3–4 days after surgery, and patients who underwent standard type 4b resections (*n* = 5) started oral feeding 7 days after surgery. Three months after surgery, patients without a clinical history of previous head and neck radiotherapy/open surgery who underwent type III, IVa, and modified IVb resections showed significantly better swallowing compared to patients who underwent standard type IVb resection: grade 4–6 impairment of swallowing in 8 and 66.7% of cases, respectively (*p* = 0.006072); patients with a clinical history of previous head and neck radiotherapy/open surgery who underwent type III, IVa, and modified IVb resections showed not statistically significant better swallowing compared to patients who underwent standard type IVb resection: grade 4–6 impairment of swallowing at 3 months in 16.7% and 50% of cases, respectively (*p* = 0.23568). Transoral CO_2_ laser supraglottic laryngectomy is an oncologic sound alternative to traditional open neck surgery and chemo-radiotherapy. Recovery of swallowing is significantly worsened after total resection of the arytenoid. Modified type IVb procedure leaving intact, when possible, the inferior third of the arytenoid and consequently the glottic competence, improves functional outcome.

## Introduction

Squamous cell carcinoma (SCC) arising from the vestibule, the false cords, and the epiglottis with or without limited extension to the pyriform sinus and to the arytenoid may be treated through an endoscopic and organ-preservation approach that allows for the complete removal of the lesion without the need for “open surgery”.

Currently, endoscopic CO_2_ laser supraglottic laryngectomy (ESL) targets mainly Tis, T1, and T2 tumors involving the epiglottis, the false vocal cords, and the aryepiglottic folds but also selected cases of T3 and T4 supraglottic lesions (1–3).

The choice of the most adequate treatment shouldn't be based only on the oncologic results of a procedure, but also quality of life issues have to be considered. Vocal function is generally not significantly impaired by supraglottic resection except during the wound healing period soon after the surgical procedure, while partial or complete resection of supraglottic structures can be burdened by post-operative aspiration during swallowing (4). The need for a nasogastric feeding tube, tracheostomy and/or a percutaneous endoscopic gastrostomy (PEG) are the main criteria used to evaluate early and long-term post-operative functional impairment (5, 6), but recovery of swallowing could be impaired by different degrees of aspiration, and consequently, feeding intake limitations can be observed even in patients without tracheostomy and/or PEG.

Incomplete airway closure has been mainly associated with arytenoid resection (1, 4) (corresponding to type IVb supraglottic laryngectomy according to the European Laryngological Society Classification) (3) and has always been considered with caution while planning the treatment strategy as already observed for type Vb cordectomy.

The present study analyzed the results of a single surgeon's experience (RP) with transoral CO_2_ laser standardized supraglottic laryngectomy, with the aim of evaluating the functional results after different types of resections. Oncologic outcomes, the role of neck dissection and of the adjuvant radiotherapy were also evaluated.

## Methods

This is a retrospective clinical study of 42 consecutive patients (mean age of 61.8 years; range of 43–84 years) treated for supraglottic SCC by ESL with a CO_2_ laser and reviewed from November 2010 to September 2017. The present study was approved by the Ethics Committee “Commissione del Comitato Etico Indipendente della A.O.U. di Cagliari,” under protocol number NP/2018/895.

Eleven patients (26.2%) had a clinical history of previous surgical treatment for other head and neck malignancies: 5 oral cavity SCC (surgical treatment was associated in 2 cases with adjuvant radiotherapy), 3 open horizontal laryngectomies (surgical treatment was associated in 1 case with adjuvant radiotherapy), and 3 transoral glottic surgery (1 type Va, and 2 type III cordectomies). Two patients underwent ESL after failure of chemo-radiotherapy, and 2 patients underwent ESL after platinum-based neoadjuvant chemotherapy (3 cycles of cisplatinum associated with 5-fluorouracil).

Pre- and intra-operative work-up included objective local examination by flexible and rigid endoscopy coupled in 2013 with narrow band imaging (NBI), panendoscopy with 0 and 30° rigid scopes associated with IMAGE 1S (Storz, Germany) and enhanced contact endoscopy (ECE) (7). Preoperative imaging included computed tomography (CT) and/or magnetic resonance (MR) with contrast medium of the head neck and CT scan with contrast medium of the chest in order to better assess the lymph node involvement, invasion of the pre-epiglottic and paraglottic spaces, contiguous subsites, and distant metastases.

Procedures were performed under general anesthesia through orotracheal or tracheal intubation with a Mallinckrodt tube Athlone, Ireland (I.D. 5.0–7.0 mm). To obtain complete exposure of the larynx, pharyngo-laryngoscopy was carried out using an adjustable bivalve Storz® (Tuttlingen, Germany) pharyngo-laryngoscope (Weerda type). Surgical procedures were classified according to the European Laryngological Society (3). Patients with limited involvement of the antero-medial aspect of the arytenoid underwent modified type IVb procedure extended to the superior two-thirds of the arytenoid.

The following microscopes were used during the surgical procedure: Zeiss Universal S2 or Zeiss S21 (Jena, Germany) with 400 mm focal lens coupled with an Acupulse (Tel Aviv, Israel) CO_2_ laser with an Acublade (Tel Aviv, Israel) focusing system, with which it was possible to obtain a 150-micron spot. The super-pulse mode was used at 10 watts. Blood vessels larger than 0.5–1 mm in diameter were coagulated with bipolar forceps or clamped with microclips. With respect to the cleavage plane, the laser was always used in cutting mode and never in vaporization mode in order to obtain precise histological information of the entire specimen.

Frozen sections for the intraoperative evaluation of surgical margins were not routinely used in type I-II ESL. Surgical specimens were sent to a dedicated pathologist after marking one designated edge with black ink in the “en bloc” excisions. In “multibloc” procedures, all surgical margins (superficial and deep) were assessed, and the distance between tumor and margins was measured. A surgical margin with more than 1 mm of healthy tissue around the tumor was considered as “negative,” “close” with 1 mm or less, and “positive” when the tumor was present at the level of one or more superficial and/or deep resection margins (2).

Patients submitted to neck dissection were treated with synchronous or within 30 days delayed neck procedure unilaterally or bilaterally according to the tumor relationship with the midline and the availability of the definitive histology. A wait-and-see policy for the neck was chosen in selected frail patients and in patients with cT1N0 and small cT2N0 lesions (i.e., T2 with a volume comparable to that of a T1 but encroaching on two adjacent subsites, as in the case of a tumor centered between the supra- and infra-hyoid epiglottis). All patients with clinically positive nodes were always treated by therapeutic unilateral or bilateral neck dissection performed simultaneously with the transoral procedure.

Temporary tracheostomy was considered according to the extent of the resection, the simultaneous neck dissection, and comorbidities.

A naso-gastric feeding tube was not considered necessary after type I–II resections.

Based on definitive pathologic findings, tumors were re-staged according to the 8th edition of the American Joint Committee on Cancer (AJCC) system (8).

Patients with positive margins were submitted after counseling to endoscopic enlargement usually at the time of neck dissection (when delayed).

Patients with neoplastic perineural and vascular invasion, multiple positive lymph nodes, and/or extracapsular spread underwent adjuvant radiotherapy/chemo-radiotherapy (9).

After healing the surgical wound, voice and swallowing rehabilitation and regular follow-up were planned according to National Comprehensive Cancer Network (NCCN) guidelines (10).

Swallowing was evaluated on the basis of the Swallowing Performance Status Scale (SPS) reported by the Multinational Association of Supportive Care in the Cancer/International Society of Oral Oncology (11):

- Grade 1: normal
- Grade 2: patients show functional limits but is able to eat a regular diet without modifications or swallowing precautions.
- Grade 3: patients show mild impairment or mild dysfunction that requires a modified diet without need for therapeutic swallowing precautions.
- Grade 4: patients show mild-to-moderate impairment and require a modified diet and therapeutic precautions to minimize aspiration risk.
- Grade 5: patients show moderate impairment or moderate dysfunction with aspiration seen on exam that requires a modified diet and swallowing precautions to minimize aspiration events.
- Grade 6: patients show moderate-to-severe dysfunction with aspiration seen on exam that requires a modified diet and swallowing precautions to minimize aspiration events, or supplemental enteral feeding support.
- Grade 7: patients show severe impairment or severe dysfunction with significant aspiration or inadequate transit to the esophagus that requires primary enteral feeding support.

Objective analysis of the dysphagia included a swallowing test performed under fibrolaryngoscopy, with stained food and fluid, and videofluoroscopy to objectivate subclinical laryngo-tracheobronchial aspiration.

Patients included in the present study were followed up from the date of surgery until, when possible, February 2018.

Survival probabilities over time were estimated using Kaplan-Meier curves and Cox proportional hazard models. Statistical analyses were performed using GraphPad Prism software (GraphPad, San Diego, CA, USA). Time-related study endpoints were overall survival (OS), disease-specific survival (DSS), local recurrence free survival, nodal recurrence free survival, and overall laryngeal preservation rate (OLP). Log-rank (Mantel-Cox) tests were applied to compare survival rates between the different patient groups (pT category, patients with or without previous head and neck radiotherapy/open surgery, and cN0/cN1 patients who underwent primary neck dissection vs. patients who underwent close follow-up). The Pearson *X*^2^ test was applied to compare categorical variables. A *p*-value < 0.05 was considered significant.

Functional results were evaluated differentiating two groups of patients: those with a clinical history of previous head and neck radiotherapy/open surgery that could have impaired swallowing before endoscopic management (*n* = 10) vs. naive patients (*n* = 32); patients never treated before with head and neck radiotherapy/open surgery (naive). Patients previously treated endoscopically for glottic tumors (*n* = 3) and those who underwent neoadjuvant therapy (*n* = 2) were considered as naive patients because the endoscopic management of glottic tumors has minimal effects on swallowing (none of the patients included in the present study was treated by a cordectomy extended to the arytenoid or other supraglottic areas), and because neoadjuvant therapy was part of the conservative approach.

The literature review was based on an extensive MEDLINE search using “supraglottic cancer,” “transoral,” “endoscopy,” “CO_2_ laser,” “microsurgery,” and “functional results” as keywords.

## Results

This study included 33 males (mean age of 63.9 years; range of 44–84 years) and 9 females (mean age of 55 years; range of 43–74 years).

Daily alcohol consumption was reported by 30 patients (71.4%), and daily tobacco consumption was reported by 32 patients (76.2%).

The following resections were performed: 2 type I, 2 type IIa, 2 type IIb, 3 type IIIa, 12 type IIIb, 13 type IVa, 3 modified type IVb, and 5 type IVb supraglottic laryngectomies. Twenty-one patients (50%) staged as cT1N0 in 2 cases, cT2N0 in 2 cases, cT3N0 in one case, cT1N1 in one case, cT2N1 in 13 cases, cT3N1 in one case, and cT3N2b in one case underwent primary neck dissection (16 simultaneously and 5 within 30 days after the transoral procedure), 7 patients (16.7%) with a clinical history of neck dissection for previous head and neck malignancies (bilateral in 5 cases and unilateral in 2 cases) did not undergo neck dissection, and 14 patients (33.3%) underwent a close wait-and-see policy of the neck status.

In 23 cases (54.8%), temporary tracheostomy was performed.

One patient experienced post-operative bleeding that was controlled under general anesthesia.

Patients who underwent type I, IIa and IIb resections (*n* = 6) started oral feeding the day after surgery, while a naso-gastric feeding tube was considered necessary in the other 36 patients (85.7% of the series, mean time of persistence of the naso-gastric feeding tube of 5.9 days, range of 3–18 days). Patients who underwent type IIIa, IIIb, IVa, and modified IVb resections (*n* = 31) started oral feeding 3–4 days after surgery, and patients who underwent standard type IVb resections (*n* = 5) started oral feeding 7 days after surgery. The need for a nasogastric feeding tube was longer in patients who underwent standard type IVb resections (19 days vs. 9 days in patients who underwent type IVa and modified IVb resections). No patient had percutaneous endoscopic gastrostomy (PEG). The time of hospitalization, permanence of tracheostomy and of naso-gastric feeding tube are detailed in Table 1.

**Table 1:** Mean hospitalization time, need for temporary tracheostomy, and need for naso-gastric feeding tube for each type of transoral supraglottic laryngectomy/group of patients.

	**Mean hospitalization time (days)**	**Permanence of temporary tracheostomy (days)/Number of patients who underwent tracheostomy**	**Permanence of naso-gastric feeding tube (days)**
All patients (*n* = 42)	9.8	9.7/23	9.6
Type I ESL (*n* = 2)	4	3/1	–
Type II ESL (*n* = 4)	6	3/1	–
Type III ESL (*n* = 15)	10	9/9	7
Type IVa ESL (*n* = 13)	10	10/8	9
Type IVb mod ESL (*n* = 3)	10	10/1	9
Type IVb ESL (*n* = 5)	14	15/3	19
Naive patients (*n* = 32)	9.6	9.6/19	7.7
Type I ESL (*n* = 1)	3	–	–
Type II ESL (*n* = 3)	6.5	3/1	–
Type III ESL (*n* = 14)	10	9/9	7
Type IVa ESL (*n* = 9)	10	10/6	8.6
Type IVb mod ESL (*n* = 2)	10.5	10/1	9
Type IVb ESL (*n* = 3)	11.3	14/2	17.5
Patients with previous treatments (*n* = 10)^*^	10.4	10/4	10
Type I ESL (*n* = 1)	5	3/1	–
Type II ESL (*n* = 1)	4.5	–	–
Type III ESL (*n* = 1)	9	–	7
Type IVa ESL (*n* = 4)	10	10/2	10
Type IVb mod ESL (*n* = 1)	9	–	9
Type IVb ESL (*n* = 2)	18	17/1	22

* *Patients with a clinical history of previous head and neck radiotherapy/open surgery*.

Thirty-four procedures (81%) were performed in free margins of resection. Four procedures (9.5%) showed close margins: one patient underwent surgical enlargement (definitive histology of the enlargement did not find residual tumor), and 3 were submitted to a wait-and-see policy; among them, one patient experienced recurrence on primary and underwent total laryngectomy. One procedure (2.4%) was performed with margins involved by mild dysplasia, and the patient underwent surgical enlargement (histology showed residual mild dysplasia removed with free margins). In 3 cases (7.1%), the excision was performed with positive deep margins: two patients underwent surgical enlargement (no evidence of residual disease after definitive histology), and one patient underwent radiotherapy.

The pathologic TNM classification according to the AJCC system (8) was as follows: 9 pT1cN0, 2 pT1N0, one pT1N1, 7 pT2cN0, one rypT2cN0, 9 pT2N0, 4 pT2N1, 2 ypT2N1, 2 pT3cN0, 2 rypT3cN0, one pT3N1, and 2 pT3N2b.

Eight patients (19%) underwent adjuvant radiotherapy (Table 2).

**Table 2:** Patients who underwent adjuvant radiotherapy.

**N/Sex/Age (years)**	**cTNM**	**Type of ESL**	**pTNM**	**Margins**	**Surgical enlargement**	**Vascular invasion**	**Nodal involvement**	**Dosage of radiotherapy**	**Associated chemotherapy**	**Outcomes**
1/M/67	cT2 N1	IVa	pT2 N1	Involved (deep margin)	Yes (No residual tumor)	Yes	1 ipsilateral lymph node (extracapsular spread)	66 Gy	No	NED after 1 year of follow-up
2/F/70	cT2 N0	IVa	pT2 cN0	Involved (deep margin)	No	No	No	66 Gy	8 cycles of CISPLATIN	NED after 5.3 years of follow-up
3/M/63	cT1 N1	IIIb	pT2 N1	Free	No	No	1 ipsilateral lymph node	54 Gy	12 cycles of CISPLATIN	DOC after 4.2 years of follow-up
4/F/52	cT2 N2b	IVa	pT3 N2b	Involved (deep margin)	Yes (No residual tumor)	No	Multiple (2) ipsilateral lymph nodes	66 Gy	No	NED after 5.8 years of follow-up
5/M/63	cyT2 N2c^**^	IIIb	ypT2 N1	Free	No	No	1 ipsilateral lymph node	54 Gy	No	NED after 5 years of follow-up
6/F/57	cT2 N0^*^	IVa	pT3 cN0	Free	No	No	No	54 Gy	No	NED after 3.5 years of follow-up
7/M/61	cT2 N2a	Modified IVb	pT3 N2b	Close (deep margin)	Yes (No residual	No	1 ipsilateral lymph node (extracapsular spread)	66 Gy	No	DOC after 1.6 years of
8/M/80	cT1 N0	IVa	PT2 N1	Free	No	No	1 ipsilateral lymph node	54 Gy	No	DOC after 0.7 years of follow-up

* *Patient with a clinical history of previous head and neck open surgery*.

** *Patient who previously underwent neoadjuvant chemotherapy*.

Three months after surgery, naive patients who underwent type III, IVa, and modified IVb resections showed significantly better swallowing compared to patients who underwent standard type IVb resection: grade 4–6 impairment of swallowing in 8 and 66.7% of cases, respectively (*p* = 0.006072), as shown in Table 3; one patient was submitted to a formal type IVb ESL and experienced two episodes of aspiration pneumonia but refused the proposed PEG or total laryngectomy. Patients of the second group who underwent type III, IVa, and modified IVb resections showed not statistically significant better swallowing compared to patients who underwent standard type IVb resection: grade 4–6 impairment of swallowing at 3 months in 16.7 and 50% of cases, respectively (*p* = 0.23568), as shown in Table 4.

**Table 3:** Swallowing results according to the Swallowing Performance Status Scale of all patients without history of previous head and neck radiotherapy/open surgery.

**Degrees of dysphagia**	**All series**	**Type I resection**	**Type II resection**	**Type III resection**	**Type IVa resection**	**Type IVb modified resection**	**Type IVb resection**
Grade 1	15	1	3	8	3		
Grade 2	8			4	3 (1)^*^	1	
Grade 3	5			1 (1)^*^	2^*^	1^*^	1
Grade 4	2			1^*^	1^*^		
Grade 5	1						1
Grade 6	1						1
Grade 7							
Total	32 (7)^*^	1	3	14 (2)^*^	9 (4)^*^	2 (1)^*^	3

* *Number of patients who underwent adjuvant radiotherapy*.

**Table 4:** Swallowing results according to the Swallowing Performance Status Scale of all patients who previously underwent head and neck radiotherapy/open surgery.

**Degrees of dysphagia**	**All series**	**Type I resection**	**Type II resection**	**Type III resection**	**Type IVa resection**	**Type IVb modified resection**	**Type IVb resection**
Grade 1	2	1			1		
Grade 2	3		1		2 (1)^*^		
Grade 3	3			1		1	1
Grade 4	2				1		1
Grade 5							
Grade 6							
Grade 7							
Total	10 (1)^*^	1	1	1	4 (1)^*^	1	2

* *Number of patients who underwent adjuvant radiotherapy*.

The mean follow-up was 3.4 years (range of 9 months to 6 years).

During the 5-year follow-up, 11 patients died from other causes (among them, 2 patients died from other causes within one year after surgery): pulmonary carcinoma in 5 cases, liver cancer in one case, hearth failure in one case, stroke in 2 cases, pulmonary embolism in one case, and unknown in one case), and 2 patients died from the disease.

Nine patients (21.4%) experienced recurrence of the disease as shown in Table 5 (4 on ipsilateral neck, one on contralateral neck, one on ipsilateral neck and after one year on primary and three on primary) and underwent total laryngectomy (*n* = 3), neck dissection (*n* = 6), and open horizontal laryngectomy (*n* = 1). Three patients underwent adjuvant radiotherapy after rescue therapy. Kaplan-Meier analysis revealed 5-year disease-specific survival, local-relapse-free survival, nodal-relapse-free survival, overall laryngeal preservation and overall survival of 93.1, 90.5, 83, 90.7, and 64.9%, respectively (Table 6).

**Table 5:** Recurrences.

**N/Sex/Age (years)**	**Previous head and neck surgery**	**Previous CHT/RT**	**cTNM**	**Type of ESL**	**pTNM/margins**	**Adjuvant therapy**	**Site of recurrence/Time of recurrence**	**Salvage treatment/rpTNM**	**Second recurrence**	**Treatment of second recurrence/rpTNM**	**Status/Follow-up**
1/M/57	No	No	cT2N0	IIIa	pT2cN0/R0	No	Ipsilat. Neck/0.4 years	Ipsilat. ND + RT (66 Gy)/rpT0N2a	Primary/2 years	IIb OPHL/rypT2cN0	DOC/5.1 years
2/M/81	No	No	cT2N0	IIIb	pT2cN0/R0	No	Bilat. Neck/0.7 years	Bilat. ND/rpT0N2b	No	/	DOC/1 year
3/F/48	No	CHT-RT for cT3 supraglottic SCC	ycT3N0	IVb	ypT3cN0/R0	No	Ipsilat. Neck/0.3 years	Ipsilat. ND/rypT0N2b	No	/	NED/5.2 years
4/F/43	No	CHT-RT for cT3 supraglottic SCC	yCT3N0	IIIb	pT3cN0/R1	No	Primary/1.2 years	TL/rypT3cN0	No	/	NED/5 years
5/F/72	No	No	cT1N1	IVa + Ipsilat. ND	pT1N1/R0	No	Contralat. Neck/1.8 years	Contralat. ND + RT (66 Gy)/rpT0N2a	No	/	NED/5.2 years
6/M/55	Head and Neck surgery for pT4aN1 oral SCC	RT (54 Gy)	cT2N0	IVa	ypT2cN0/R0	CHT	Ipsilat. Neck/1 year	Ipsilat. ND + CHT/rypT0N2a	Ipsilat. Neck/2 years	CHT	DOD/2.3 years
7/M/69	Head and Neck surgery for pT1N2 oral SCC	RT (54 Gy)	cT3N0	IVa	ypT3cN0/R0	No	Primary/0.4 years	TL + CHT/rypT4acN0	Primary/1.2 years	CHT	DOD/2.5 years
8/M/53	Head and Neck surgery for pT1N2b laryngeal SCC	CHT-RT (66 Gy)	rycT2N0	IVb	rypT2cN0/R0	CHT	Primary/2.7 years	TL/rypT3cN0	No	/	NED/3.6 years
9/M/66	Head and Neck surgery for pT2N0 laryngeal SCC	No	rcT1N0	I	rpT1cN0/R0	No	Ipsilat. Neck/0.2 years	Ipsilat. ND + RT (66Gy/rypT0N3	No	/	DOC/1.1 years

*CHT: chemo-radiotherapy; RT: radiotherapy; ND: neck dissection; TL: total laryngectomy; NED: no evidence of disease; DOD: died of disease; DOC: died of other causes*.

**Table 6:** 5-year survival outcomes.

	**Disease-specific survival**	**Local recurrence-free survival**	**Nodal recurrence-free survival**	**Overall laryngeal preservation**	**Overall survival**
All series (*n* = 42)	93.1% SE: 4.7	90.5% SE: 6%	83% SE: 6.3%	90.7% SE: 5.2%	64.9% SE: 8.8%
T1 (*n* = 12)	100%	100%	80.1% SE: 6%	100%	70% SE: 6%
T2 (*n* = 23)	95.7% SE: 4%	91.3% SE: 5%	83.6% SE: 7%	95.7% SE: 4%	66% SE: 6%
T3 (*n* = 7)	71.4% SE: 14%	71.4% SE: 14%	85.7% SE: 13%	71.4% SE: 14%	43% SE: 14%
Naive patients (*n* = 32)	100%	95.2% SE: 4.6%	87.8% SE: 6.3%	100%	64.6% SE:16.3%
T1 (*n* = 9)	100%	100%	85.7% SE: 13.2%	100%	75% SE: 18.9%
T2 (*n* = 20)	100%	91.7% SE: 8%	87.8% SE: 8%	100%	65% SE: 12.8%
T3 (*n* = 3)	100%	100%	100%	100%	66.7% SE: 27.2%
Previously treated (*n* = 10)	77.8% SE: 13.9%	65.6% SE: 16.4%	70% SE:14.5%	65.6% SE: 16.4%	70% SE: 14.5%
T1 (3)	100%	100%	66.7% SE: 27.2%	100%	66.7% SE: 27.2%
T2 (3)	66.7% SE: 27.2%	50% SE: 35.6%	66.7% SE: 27.2%	50% SE: 35.4%	66.7% SE: 27.2%
T3 (4)	75% SE: 21.7%	50% SE: 25%	75% SE: 21.7%	50% SE: 25%	75% SE: 21%

*SE: standard error*.

The Cox univariate analysis showed that patients with a clinical history of previous head and neck radiotherapy/open surgery (*n* = 10) experienced statistically significant worse local recurrence free survival, and OLP compared to naive patients (*n* = 32): 65.6 vs. 95.2% (*p* = 0.0181; hazard ratio of 9), and 65.6 vs. 100% (*p* = 0.0025; hazard ratio of 9) respectively, while they experienced worse but not statistically significant nodal recurrence free survival: 70 vs. 87.8% (*p* = 0.1603; hazard ratio of 7.7).

The Cox univariate analysis showed that cN0 patients who did not undergo primary neck dissection (*n* = 21) experienced lower but not statistically significant nodal recurrence free survival compared to cN0 and cN1 patients who underwent primary neck dissection (*n* = 21): 74.7% vs. 92.9% (*p* = 0.1013; hazard ratio of 4.6).

## Discussion

Alonso first described the supraglottic laryngectomy as a conservative treatment for supraglottic tumors through an external approach (12), but the first author who described transoral CO_2_ laser surgery for the removal of a supraglottic carcinoma was Vaughan in 1978 (13). The endoscopic approach has been progressively reported as an alternative to open neck supraglottic surgery (14), allowing for a radical resection, tailored on the basis of the real extension of the tumor.

Pre- and intra-operative staging is based on the precise endoscopic evaluation of the superficial extension of the neoplasms that can be performed with image-enhanced endoscopy in the pre-operative setting, and ECE used to possibly detect synchronous satellite lesions and to spot very early pre-cancerous lesions that can progress if left untreated. As reported in literature, inadequate laryngeal exposure, arytenoid fixation, massive invasion of the pre-epiglottic space, and laryngeal framework involvement still represent the “weak points” of the endoscopic approach, reducing its application in terms of technical feasibility and local control compared to more traditional surgical and non-surgical therapies (2). Vocal cord mobility must be carefully evaluated: in supraglottic cancer the tumor can easily extent to the arytenoid with encroachment of the cricoarytenoid joint. Patients with supraglottic lesions usually undergo CT or MRI with contrast medium also to spot the pre-epiglottic space and to check for neck nodes and/or distant metastases. In our series, pre-operative evaluation failed to detect early T3 lesions as three patients previously staged as cT2 were up-staged to pT3 after definitive histology for paraglottic involvement not evident at imaging. Cancer of the “3-folds” could spread in different directions, superficially toward base of tongue, pyriform sinus, false vocal cords and epiglottis but deep invasion can involve the content of the superior paraglottic space; in this condition imaging plays an important role for the detection of the deep invasion (15), but in some instances only histology confirms its involvement, upstaging the class of tumor; this is also the most frequently area in which vascular invasion or perineural spread is detected at histology giving the indication for adjuvant therapy (9) as in our series.

Platinum-based induction chemotherapy regimens in patients with intermediate/advanced laryngeal carcinoma have been reported in literature to down-stage T3 trans glottic carcinoma by Laccourreye et al. allowing for remobilization of the fixed arytenoid cartilage who became amenable to conservative surgical approach (16). In our series platinum-based induction chemotherapy was performed in two patients with bulky T2 and although TNM staging did not change, we think that the transoral approach was facilitated by the reduction of the volume of the tumor in at least one patient. On the basis of this limited experience we are not able to state that neoadjuvant chemotherapy followed by ESL has a real role on organ-preservation strategies and more studies are needed.

Every effort should be made to obtain clear margins during the first endoscopic procedure or with a second-look surgery to reduce any over-treatment or multimodal therapies (14). Piecemeal resection allows for the better management of bulky lesions, apparently without consequences for dissemination of neoplastic cells to the neck or distant subsites (17).

In case of close margins or involved margins, the following policy was followed: a radicalization was performed within 4 weeks after surgery in 1 over 4 patients with close margins (the remaining underwent a wait-and-see policy), while 3 over 4 cases with positive margins underwent a second look with enlargement of the surgical margins (in 1 case it was coupled with the delayed neck dissection), and one patient underwent adjuvant radiotherapy (positive deep margin). Seven patients who showed close or positive margins did not experience recurrent carcinoma, while one patient with close margin (clinical history of previous chemo-radiotherapy before the ESL) addressed for a wait-and-see policy, experienced a relapse on primary after 1.2 years of follow-up and underwent total laryngectomy as a salvage procedure.

Tumors of the 3-fold area extending to neighboring subsites can be treated by extended ESL, such as type IVa resection, including the free edge of the epiglottis, the 3-fold area and the ventricular fold. In such cases, the resection may include the inner or medial wall and anterior corner of the pyriform sinus without major functional consequences. In case of extension of the neoplasm to the anterior/superior aspect of a mobile arytenoid, the arytenoid can be included in the resection (type IVb), but swallowing can be greatly impaired (1), while an endoscopic approach for patients with lesions extending to the cricoarytenoid joint should be avoided. We performed a modified type IVb resection in 3 patients, leaving intact the inferior third of the arytenoid including the vocal process, thereby maintaining glottic competence. Oncologic outcomes in these cases were not affected by such a tailored resection.

During types IIIb and IV ESL, the superior laryngeal artery was constantly ligated with metallic clips in order to reduce the risk of post-operative massive hemorrhage that has been reported as responsible for major complications (17). Minor bleeding in the present series occurred in one patient and required general anesthesia and bipolar cautery.

Kaplan-Meier analysis of the entire cohort of patients showed a 5-year local relapse-free survival of 90.5% and overall laryngeal preservation rate of 90.7%, showing that the endoscopic approach tailored to supraglottic carcinoma is an oncologic sound approach for the primary tumor. Local control after ESL and rescue therapy for each class of tumor is described in Table 6.

Since the incidence of occult metastases in patients with supraglottic carcinoma ranges from 20 to 40% of clinically negative necks (18–20), rising also for moderate to advanced carcinomas, neck dissection should be strongly considered for cN0 patients with supraglottic carcinoma (18). In our series, 8 out of the 26 cN0 patients (30.8%) showed occult metastases at histology or during follow-up, and patients who did not undergo primary neck dissection experienced lower but not statistically significant (*p* = 0.1252) nodal-relapse-free survival (74.7%) compared to cN0 and cN1 patients who underwent primary neck dissection (92.9%) despite the T stage. In our series, naive T1 showed neck metastasis in 1 case (11.1%), naive T2 in 8 cases (40%), and naive T3 in 2 cases (66.7%). As a consequence, as from the present series, neck dissection should be planned in almost all patients with supraglottic SCC, in agreement with UK guidelines (9), and a wait-and-see policy should be limited to very early primaries (Tis, small T1 of the superficial border of the epiglottis and of the ary-epiglottic fold) or in case of severe comorbidities. Sentinel node biopsy (SLNB) has been proposed for laryngeal cancer and has a very high negative predictive value (NPV), but the significance of the high NPV of SLNB outside the oral cavity is limited, and further studies are needed, especially for supraglottic carcinoma (21, 22).

After surgery, the choice among complementary radiotherapy or chemo-radiotherapy should be balanced in face of margin status, metastatic lymph nodes, pathologic risk factors and patient characteristics (9). In the present series, adjuvant radiotherapy was considered necessary in six patients with metastatic nodes, in one patient treated for a second advanced head and neck malignancy, and in one patient after endoscopic removal of bulky lesions with vascular invasion and deep margin involvement seen at histology.

As shown in Table 6, patients who previously underwent head and neck radiotherapy/open surgery (other head and neck malignancies in 8 cases and failure of chemo-radiotherapy in 2 cases) experienced a statistically significant worse local recurrence free survival and OLP rates compared to naive patients. Consequently, previous treatment for other head and neck cancer, including radiotherapy extending to the supraglottic area and recurrent supraglottic cancer, should be carefully considered before ESL; in such cases, small lesions in the supraglottis as usually spotted during follow-up for different head and neck primary tumors can be considered still good candidates for ESL.

Salvage surgery after failed CO_2_ laser transoral microsurgery in supraglottic tumors may be performed by ESL or, if necessary by all the different open partial horizontal laryngectomy (OPHL) and total laryngectomy. In our series, 4 patients (one naive patient and 3 previously treated with radiotherapy/open surgery) developed a recurrence on primary after ESL. The naive patient underwent a type IIb OPHL, while the 3 patients previously treated with radiotherapy/open surgery underwent total laryngectomy.

Five-year OLP was 100% in the group of naive patients and 65.6% in the second group of patients.

Second malignancies have been often observed in patients treated for supraglottic carcinoma (2), and in our series, 5 patients died for a second pulmonary malignancy, supporting the importance of regular chest CT during follow-up.

Temporary tracheostomy in patients undergoing endoscopic laser surgery for supraglottic lesions should be taken carefully into consideration when planning the extent of the resection in order to prevent aspiration and possible passage of massive amounts of blood in the case of post-operative hemorrhage (14). Furthermore, disorders with swallowing and breathing problems can be easily overcome by means of temporary tracheostomy. In our series temporary tracheostomy showed paramount benefit in one patient who experienced post-operative bleeding, and all patients submitted to temporary tracheostomy did not experience long-term complications.

Functional results after CO_2_ laser supraglottic laryngectomy have been considered in the literature, but few studies differentiated the long-term outcomes on the basis of the different endoscopic resections (1). ESL generally allows for shorter time for swallowing recovery, reduced tracheostomy maintenance, and lower incidence of aspiration pneumonia (18) because of the complete preservation of the sensation of the vestibule and hypopharynx by sparing the superior laryngeal nerve in the majority of the cases, of the accessory muscles that normally contribute to the craniocaudal movements of the larynx, and of an effective cough reflex that may prevent severe problems (4, 23, 24). However, major dysfunction after ESL, such as permanent tracheostomy and/or PEG, have been reported in the literature (Table 7) (1, 5, 6, 14, 25, 26). In the present series, we did not perform any PEG, and all tracheostomies were removed within 1 month from surgery. Removal of supraglottic structures can result in various degrees of swallowing problems, even in patients without tracheostomy and/or PEG. The MD Anderson Dysphagia Inventory (MDADI), a subjective dysphagia-specific quality of life scale, has been reported in the literature as a possible instrument (1, 4). In our series, the SPS reported by the Multinational Association of Supportive Care in Cancer/International Society of Oral Oncology (11) was preferred since it offers an accurate and objective assessment of the presence and severity of dysphagia and aspiration risk by combining subjective and objective data.

**Table 7:** Comparative results of functional results from different studies.

**Author and year of publication**	**No. of procedures/T stages**	**No. of early post-operative complications (%)**	**No. of permanent tracheostomies (%)**	**No. of patients who required additional procedures to avoid aspiration (%)**
Puxeddu et al. (14)	12 T1:3 T2:9	1 (8.3%)	0 (0%)	0 (0%)
Cabanillas et al. (6)	26 T1:3 T2:8 T3:15	13 (50%)	2 (7.7%)	4 (15.4%)
Bernal-Sprekelsen et al. (5)	121 T1:16 T2:41 T3:53 T4:11	**/**	2 (1.7%)	2 (1.7%)
Chiesa Estomba et al. (25)	31 T1:2 T2:15 T3:14	6 (19.4%)	2 (6.5%)	4 (13%)
Piazza et al. (1)	96 T1:28 T2:46 T3:22	10 (11%)	0 (0%)	0 (0%)
Bertolin et al. (26)	15^*^ T1:2 T2:12 T3:1	/	0 (0%)	0 (0%)

* *Patients included in the study with the analysis of the functional results*.

The resection of the epiglottis alone, even when it is completely removed, does not significantly hamper the laryngeal sphincter, which is partially preserved by the adduction of the vestibular bands and the vocal folds (1). In our experience, the resection of the epiglottis was not associated with negative functional outcomes (see Tables 3, 4) such as the resection of the pre-epiglottic content as indeed reported by Peretti et al. (2, 17).

Lateral resection of the aryepiglottic fold, mostly when extended to the arytenoid, can hinder the laryngeal competence and, more notably, facilitate the aspiration of the bolus from the pyriform sinus to the laryngeal pathway due to the inadequacy of the barrier between the two. The reduction of the sensitiveness of the upper portion of the pyriform sinus due to resection of the pharyngo-epiglottic fold and its sensitive receptors, and the section of the superior laryngeal nerve may further worse swallowing (1). In our patients, sensation of the vestibule evaluated under fibrolaryngoscopy was generally preserved as the result of the limited traumatism by the CO_2_ laser since the resection was always performed by tractioning the tissues with minimal power density, reducing the potential injury to the superior laryngeal nerve. The modification of type IVb resection is feasible if there is no major encroachment of the arytenoid or the vocal cord. Although limited to few cases, in our experience, patients who underwent resection extended only to the superior 2/3 of the arytenoid showed significantly better long-term swallowing function than patients who underwent standard type IVb resection (*p* = 0.029501) and had reduced time with naso-gastric feeding tube (9 days in patients who underwent IVb modified resections vs. 19 days in patients who underwent standard IVb procedures), highlighting that the residual arytenoid/vocal cord adduction preserves adequate glottic competence. One patient submitted to a formal type IVb ESL experienced two episodes of aspiration pneumonia but refused a proposed PEG or total laryngectomy, and the SPS staging was considered grade 6.

Although adjuvant radiotherapy could be associated with worse functional outcomes (4), our patients who underwent adjuvant radiotherapy experienced minor and not statistically significant worsening of swallowing function (*p* = 0.144061).

In conclusion, ESL is an oncologic sound alternative to traditional open neck surgery and radiotherapy and chemo-radiotherapy, and, although it requires a higher level of experience in endoscopic surgical technique, it allows to perceive a competitive organ-preservation approach with predictable functional outcomes and acceptable complication rates.

The present study, in agreement with previous studies (1, 2), confirmed that greater morbidity is seen following more extended procedures. In our opinion, the endoscopic removal of the arytenoid can be avoided in some cases, and lesions with moderate involvement of the arytenoid could be treated by a less aggressive procedure by sparing part of the inferior aspect of the arytenoid with significant improvement in swallowing recovery, while massive involvement of the arytenoid could be considered a contraindication for the endoscopic approach.

Primary elective neck dissection is not in contrast with the concept of lower morbidity of the endoscopic approach and, in the present study, increased regional control of the disease.

## Author contributions

All authors contributed to the preparation of the manuscript. FC, CM, and RP wrote the manuscript. NC, EL, CM, and GS collaborated in the collection and analysis of the clinical data. CG was in charge of the pathologic analysis. All of the authors reviewed and contributed to the present form of the manuscript.

### Conflict of interest statement

The authors declare that the research was conducted in the absence of any commercial or financial relationships that could be construed as a potential conflict of interest.
